# Community cohesion looseness in gene networks reveals individualized drug targets and resistance

**DOI:** 10.1093/bib/bbae175

**Published:** 2024-04-15

**Authors:** Seunghyun Wang, Doheon Lee

**Affiliations:** Department of Bio and Brain Engineering, KAIST, Daejeon, Republic of Korea; Department of Bio and Brain Engineering, KAIST, Daejeon, Republic of Korea

**Keywords:** systems biology, gene expression, individualized gene network, precision medicine, biomarker discovery

## Abstract

Community cohesion plays a critical role in the determination of an individual’s health in social science. Intriguingly, a community structure of gene networks indicates that the concept of community cohesion could be applied between the genes as well to overcome the limitations of single gene-based biomarkers for precision oncology. Here, we develop community cohesion scores which precisely quantify the community ability to retain the interactions between the genes and their cellular functions in each individualized gene network. Using breast cancer as a proof-of-concept study, we measure the community cohesion score profiles of 950 case samples and predict the individualized therapeutic targets in 2-fold. First, we prioritize them by finding druggable genes present in the community with the most and relatively decreased scores in each individual. Then, we pinpoint more individualized therapeutic targets by discovering the genes which greatly contribute to the community cohesion looseness in each individualized gene network. Compared with the previous approaches, the community cohesion scores show at least four times higher performance in predicting effective individualized chemotherapy targets based on drug sensitivity data. Furthermore, the community cohesion scores successfully discover the known breast cancer subtypes and we suggest new targeted therapy targets for triple negative breast cancer (e.g. KIT and GABRP). Lastly, we demonstrate that the community cohesion scores can predict tamoxifen responses in ER+ breast cancer and suggest potential combination therapies (e.g. NAMPT and RXRA inhibitors) to reduce endocrine therapy resistance based on individualized characteristics. Our method opens new perspectives for the biomarker development in precision oncology.

## INTRODUCTION

In social science, there has been much emphasis on the role of community cohesion in the determination of an individual’s health. Previous studies found that social ties reduce the risk of diseases and mortality [1–3]. Intriguingly, the concept of community cohesion could be applied between the genes as well. Underneath this lies a community structure of gene networks, a property that the genes involved in the same cellular functions actively interact with each other [4, 5]. In addition, there are growing pieces of evidence that the interactions between the genes are hindered in disease states [6–8]. Given these, considering how the genes lose their community cohesion could yield new insights into biomarker discovery.

However, till now, most of the efforts to discover the biomarkers for precision oncology mainly focused on genetic mutations and abnormal gene expressions of a single gene or a few [9–11]. Unfortunately, the single gene-based biomarkers have unveiled their inherent limitations that they are not enough to fully elucidate heterogeneity and complexity of oncogenesis. Consequently, the limited number of cancer patients take advantage of them in clinical practice. For example, only 19% of the cancer patients benefit from the actionable genetic mutations which have the corresponding treatments according to a recent survey [12].

In line with this, there is an urgent need to develop community-based biomarkers to overcome the limitations of the single-gene based biomarkers [13]. In the community-based biomarkers, each community in the gene networks is regarded as a cellular functional unit and the functionality of the corresponding community is measured by comprehensively considered multiple genes. Many studies showed that the community-based biomarkers achieve greater predictive power and reproducibility compared with the single-gene based biomarkers [13] in terms of predicting the disease states [14, 15] or prognosis [16, 17].

Despite this achievement, the current community-based biomarkers have a limitation that they are node-centric approaches, which basically averaged the expression levels of the genes present in the communities. However, even if the two genes are abnormally expressed identically, the impact on community cohesion may differ depending on how they are interacting with other genes in the community [18, 19]. For example, the genes which interact with a greater number of the genes (e.g. hub genes) would have a greater impact on the community cohesion looseness when they are abnormally expressed. Hence, rather than simply aggregating the expression levels of the genes, considering the individual characteristics shown in the community cohesion looseness in the gene networks could fill a niche in the community-based biomarker discovery (Supplementary Figure S1). Fortunately, the recent individualized gene network estimation methods [20–22] suggest possibilities to identify the individualized perturbed interactions.

Here we developed community cohesion scores which capture unique characteristics of the community cohesion looseness in the individualized gene network. To this end, we first estimated the individualized gene network by identifying individualized perturbed interactions which are deviated from co-expression patterns in normal tissues. Next, in each individualized gene network, we measured the community cohesion scores which precisely quantify the community ability to retain the normal interactions between the genes and their cellular functions. Using breast cancer as a proof-of-concept study to illustrate our method, we showed that the community cohesion scores can be used in various aspects of precision oncology by finding the communities which significantly lose their cohesions and by finding the genes which greatly contribute to the community cohesion looseness in each individual.

## MATERIALS AND METHODS

### Overview

**Figure 1:**
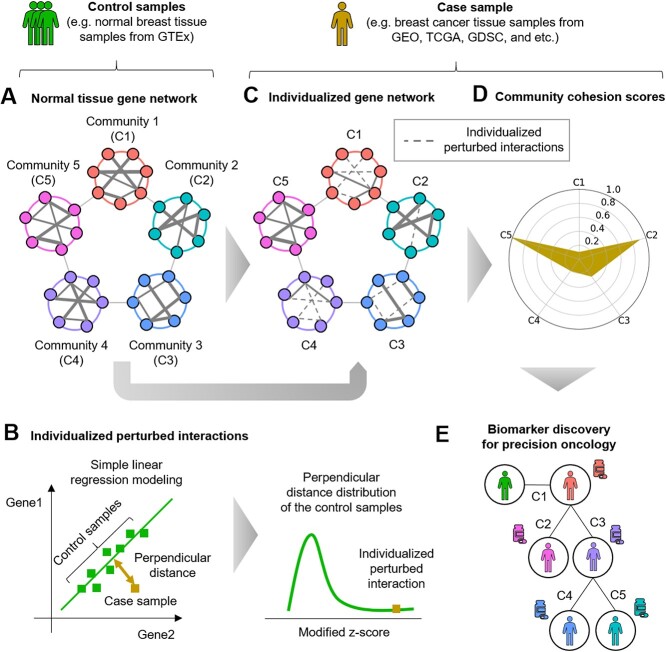
**Overall methods** (A) The normal tissue gene networks were constructed using the gene expression profiles of the control samples and the co-expressed communities were detected. (B), (C) The individualized gene network was estimated by identifying the individualized perturbed interactions deviated from co-expression patterns in the normal tissue. (D) The community cohesion scores, which precisely quantify the community ability to retain the normal interactions between the genes and their cellular functions, were used (E) to discovery biomarkers for precision oncology.

Our method requires as input a user-provided single gene expression sample (a case sample) to estimate the individualized gene networks and to measure the community cohesion scores (Figure 1). To this end, we first constructed a normal tissue gene network (tissue-specific weighted co-expression network) and found co-expressed communities using publicly available normal gene expression profiles (control samples) (Figure 1A). Next, among the interactions in each community of the normal tissue gene network, we identified the individualized perturbed interactions of the case sample, which are deviated from the co-expression patterns in the normal tissue gene network (Figure 1B). Then, we estimated the individualized gene network by removing the individualized perturbed interactions from the normal tissue gene network (Figure 1C). The final output of our method is community cohesion scores which precisely quantify the decreased cellular functionality of each community in the individualized gene network of the case sample (Figure 1D). The community cohesion scores are used to discover therapeutic and prognostic biomarkers to predict individualized drug targets and resistance for precision oncology (Figure 1E).

### Constructing the normal tissue gene network and detecting the communities

We constructed the normal tissue gene network which can represent biological systems of the normal tissue. We used the normal gene expression profiles (control samples) obtained from publicly available databases (e.g. GTEx [23]) and weighted gene co-expression network analysis (WGCNA) [24, 25] to construct the normal tissue gene network (Figure 1A and Supplementary Figure S2). First of all, we divided the control samples into a training set (80%) and a test set (20%). Using the training set, we determined parameters (pickSoftThreshold) to calculate adjacency matrix (adjacency) and weight matrix (TOMsimilarity). Then, we found the co-expressed communities which are highly interconnected with each other using the weight matrix as input features of hierarchical clustering (hclust, cutreeDynamic) [5]. Moreover, permutation test was conducted to ensure the significance of each community using the test set (communityPreservation). We only considered the communities that are significantly preserved in the test set (preservation Zsummary >10). In addition, as the WGCNA provides the weights of all gene pairs even though they are close to zero (fully connected), we remained only top 10% interactions of the largest weights in each community. It enables to sort out more reliable gene interactions [24] and make the comparable size of networks with interactomes which are frequently used in network biology [26–28]. 

### Modeling the correlations of interactions through simple linear regression and measuring perpendicular distance distributions of the control samples

To estimate individualized perturbed interactions, we first made perpendicular distance distributions of the control samples for each interaction in the normal tissue gene network (Figure 1B). To this end, we performed modeling of the correlations of each interaction (*X, Y*) through simple linear regression models and the control samples (Supplementary Figure S5).


(1) \begin{equation*} \hat{Y}={\beta}_0+{\beta}_1X \end{equation*}

Then, using the gene expression levels of gene *X* and *Y* (${x}_i,{y}_i$) in the control sample *i,* we measured the perpendicular distance (${d}_i$) which refers to the distances between the actual gene expression levels and the simple linear regression models.


(2) \begin{equation*} {d}_i=\frac{\left|{\beta}_1{x}_i-{y}_i+{\beta}_0\right|}{\sqrt{\beta_1^2+1}} \end{equation*}

By collecting these perpendicular distances of all control samples, we made the distributions of every interactions. Then, median ($\overset{\sim }{d}$) and median absolute deviation $(MAD$) were measured to characterize each distribution [29] (Supplementary Figure S5). We used Python library scipy (version 1.6.2) to model the simple linear regression models and measure median and median absolute deviation of the perpendicular distances.

### Estimating the individualized gene network

Next, we estimated the individualized gene network using a single gene expression sample (a case sample) by identifying individualized perturbed interactions deviated from co-expression patterns of the normal tissue. Using the gene expression levels of gene *X* and *Y* (${x}_j,{y}_j$) in the case sample *j*, we measured the perpendicular distance $\left({d}_j\right)$ which refers to the difference between the actual gene expression levels and the simple linear regression modeled with the control samples in the previous section (Supplementary Figure S5).


(3) \begin{equation*} {d}_i=\frac{\left|{\beta}_1{x}_j-{y}_j+{\beta}_0\right|}{\sqrt{\beta_1^2+1}} \end{equation*}

Then, to evaluate the significance of the perpendicular distance of the case samples compared with the distributions estimated using the control samples, we measured modified z-scores of the perpendicular distance of the case sample which are less affected by outliers than traditional z-score.


(4) \begin{equation*} Modified\ {zscore}_j=\frac{d_j-\tilde{d}}{1.486\ast MAD} \end{equation*}

The interactions were considered as individualized perturbed interactions if *P*-value is statistically significant in one-tailed tests (*P*-value <0.001). Finally, the individualized gene network was estimated by removing these individualized perturbed interactions from the normal tissue gene network (Figure 1C). We used Python library networkx (version 2.5.1) to for this procedure.

### Measuring the community cohesion scores

To more accurately quantify the impact of the individualized perturbed interactions on community cohesion looseness in the individualized gene network, we used network efficiency. The network efficiency is a measure of the global complex network capacity to deliver information among nodes and allows a precise quantitative evaluation of the weighted network functioning [30–32]. We applied the network efficiency to each community because we regarded each community as a cellular functional unit. The network efficiency of *k*th community in each individualized gene network was calculated as follows (Supplementary Figure S6):


(5) \begin{equation*} {Network\ efficiency}_k=\frac{1}{N\left(N-1\right)}\sum_{X\ne Y\in{G}_k}\frac{1}{d\left(X,Y\right)} \end{equation*}

where ${G}_k$ and $N$ are the set and the number of nodes in *k*th community, respectively. $d\left(X,Y\right)$ is the length of the weighted shortest path between node $X$and $Y$. If the two nodes are disconnected in the network, $d\left(X,Y\right)$ was set as 0. The weighted shortest path is computed as the minimum sum of the inverse interactions weights to travel among nodes [33].


(6) \begin{align*}& {Community\ cohesion\ score}_k=\nonumber\\&\frac{{Network\ efficiency}_k\ in\ the\ individualized\ gene\ network}{\ {Network\ efficiency}_k\ in\ the\ normal\ tissue\ gene\ network} \end{align*}

Finally, the community cohesion score of *k*th community was defined as the decreased ratio of ${Network\ efficiency}_k$ in the individualized gene network compared with the ${Network\ efficiency}_k$ of the normal tissue gene network (equation 6 and Supplementary Figure S6). Consequently, each case sample has the same number of community cohesion scores with the number of communities in the normal tissue gene network (Figure 1D). We used Python library networkx (version 2.5.1) to calculate the network efficiency.

### Measuring the connectivity loss of the druggable genes in each individualized gene network

To identify the gene that greatly contributes to the community cohesion looseness in each individualized gene network, we measured connectivity loss of the druggable genes. The connectivity loss score of each druggable gene was defined as the weight summation of individualized perturbed interactions in each individualized gene network divided by the summation of interaction weights in the normal tissue gene network (equation 7).


(7) \begin{equation*} {Connectivity\ loss\ score}_x=\frac{\sum_{m\in{e}_x}{w}_m}{\sum_{m\in{E}_x}{w}_m} \end{equation*}

where ${E}_x$ and ${e}_x$ are the set of total interactions of gene$x$ in the normal tissue gene network and the individualized perturbed interactions of gene $x$ in each individualized gene network, respectively. In other words, ${e}_x$ is the subset of ${E}_x$ and ${w}_m$ indicates the weights of the *m*th interactions. If the connectivity loss of two genes are identical, the individualized therapeutic targets are prioritized by the connectivity of the druggable genes.

## RESULTS

We used breast cancer as a proof-of-concept study to illustrate our method. We constructed the normal breast tissue gene network using 459 control samples (gene expression samples of normal breast tissue) obtained from GTEx [23] (data processing details in Supplementary Methods). We discovered 27 co-expressed communities (BrC1–BrC27) and the 23 communities were highly preserved ranging in size between 59 and 899 genes after the remaining interactions with the largest weights in the normal breast tissue gene network (Supplementary Figure S2 and Supplementary Tables S1–3). After gene expression normalization to remove batch effects (Supplementary materials and Supplementary Figures S3 and S4), we estimated the individualized gene networks and measured the 23 community cohesion scores of each of the 685 case samples (113 normal breast tissue and 572 breast cancer tissue samples, Supplementary Figure S7), 134 case samples (50 normal breast tissue and 84 breast cancer tissue samples), 181 case samples (181 tamoxifen-treated ER+ breast cancer tissue samples) and 50 case samples (50 breast cancer cell-line samples) obtained from TCGA BRCA [34], GSE58135 [35], GSE6532 [36] and GDSC [37], respectively (data processing details in Supplementary Methods). Using the 23 community cohesion scores of the case samples, we successfully prioritized the individualized effect chemotherapy drugs using the community cohesion scores of the case samples. In addition, we demonstrated that the community cohesion scores can be used to discover subtype-specific therapeutic targets. In addition, we showed that the community cohesion scores can suggest potential molecular mechanisms of the individualized drug resistance.

### The breast cancer samples show the most decreased community cohesion scores in the cell cycle-related community

**Figure 2:**
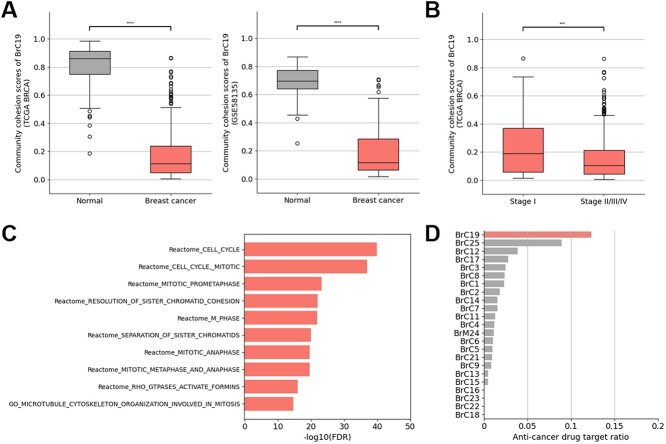
**Community cohesion scores as diagnostic biomarkers to distinguish bewteen the normal breast and breast cancer tissue samples** The community cohesion scores of the BrC19 were compared between the normal breast tissue samples and the breast cancer tissue samples using (A) TCGA BRCA and (B) GSE58135 datasets. (B) The community cohesion scores of the BrC19 were compared between the breast cancer tissue samples of stage 1 versus the samples of stage 2–4. (C) The bar plot showed the results of gene-set enrichment analysis of the genes belonged to the BrC19. (D) The ratio of anti-cancer drug targets in each community was represented as a bar plot.

We briefly confirmed the robustness of community cohesion scores through greater prediction performance and reproducibility than the single-gene based biomarkers and the node-centric community-based biomarkers in breast cancer classification using the normal breast tissue and breast cancer tissue samples of TCGA BRCA (training and validation set) and GSE58135 (independent test set) (Supplementary Methods and Supplementary Figure S8). We discovered that the community cohesion scores of the BrC19 (73 genes and 1002 interactions) were the most important features to distinguish the breast cancer tissue samples from normal breast tissue samples according to the feature importance of the logistic regression models. We also observed that the community cohesion scores of the BrC19 significantly decreased in the breast cancer tissue samples than the normal breast tissue samples using TCGA BRCA (Wilcoxon rank-sum test, *P*-value = 2.72E-60) and GSE58135 datasets (Wilcoxon rank-sum test, *P*-value = 3.62E-19) (Figure 2A). In addition, we confirmed that they also decreased according to the progression of the breast cancer by comparing the community cohesion scores of stage 1 and stage 2–4 samples (Figure 2B, Wilcoxon rank-sum test, *P*-value = 1.38E-4). In addition, we observed that BrC19 is associated with cell cycle-related terms (Figure 2C, Supplementary Methods and Supplementary Table S4). Given that the dysfunction of cell cycle is the hallmarks of cancer, these results highlight that the community cohesion scores successfully represent the individual disease states and the molecular mechanisms related to the diseases.

### The community cohesion scores can be used as therapeutic markers to prioritize individualized effective chemotherapy drugs

**Figure 3:**
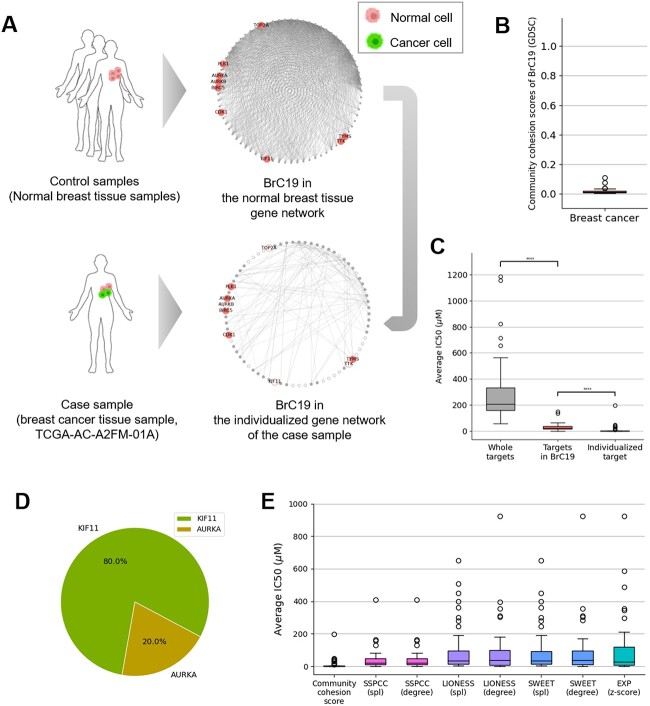
**Community cohesion scores as therapeutic biomarkers to prioritize the individualized chemotherapy targets** (A) The BrC19 of the normal breast tissue gene network was plotted. The nine druggable genes were denoted with gene symbols. The BrC19 in the individualized gene network of the breast cancer tissue sample (TCGA-AC-A2FM-01A) is plotted. The druggable genes which lose all connectivity are represented as empty circle in each individualized gene network. The human and cell figures were created with BioRender.com (B) The community cohesion scores of BrC19 in GDSC (50 breast cancer cell-line samples) were plotted. (C) The average IC50 values of all anti-cancer drugs, the anti-cancer drugs targeting the druggable genes present in the BrC19, and the anti-cancer drugs targeting the individualized therapeutic targets in the BrC19 were plotted. (D) The ratio of the individualized therapeutic targets in the BrC19 of the 50 breast cancer cell-line samples was plotted. (E) The average IC50 values were compared between when the individualized therapeutic targets are prioritized by the community cohesion scores, the previous individualized gene network construction methods and the gene expression profiles.

In addition, we obtained target gene information of approved and experimental anti-cancer drugs obtained from GDSC (542 compounds associated with 329 therapeutic targets) and measured the ratio of anti-cancer drug targets in each community. As a result, we observed that the BrC19 contains the highest ratio of anti-cancer drug targets (12.3%, Figure 2D). Based on this result, we hypothesized that the community cohesion scores could be used as therapeutic biomarkers to prioritize the individualized effective chemotherapy drugs, which target cell-cycle related biological pathways. For example, according to the therapeutic target information of GDSC, we observed that there are nine druggable genes (AURKA, AURKB, BIRC5, CDK1, KIF11, PLK1, TOP2A, TTK and TYMS) in the BrC19 (Figure 3A) and they are targeted by 28 FDA-approved and experimental anticancer drugs (Supplementary Table S6). We hypothesized that targeting these druggable genes present in the BrC19 could be more effective than targeting the other druggable genes.

Furthermore, we hypothesized that, even though all breast cancer tissue samples show the most decreased community cohesion scores in the BrC19, the druggable genes that greatly contribute to the community cohesion looseness of the BrC19 could differ in each case sample. For example, breast cancer tissue sample (TCGA-AC-A2FM-01A) has the 911 individualized perturbed interactions among the 1002 interactions in the BrC19 and shows the greatly decreased community cohesion scores (0.170) of the BrC19. Especially, four druggable genes lose all its connectivity (AURKB, KIF11, TOP2A and TTK) in the individualized gene network of the breast tissue sample (Figure 3A). Hence, we next turned to confirm that targeting these individualized therapeutic targets that greatly contribute to the community cohesion scores (high connectivity loss score) in each individualized gene network could be more effective in terms or prioritizing the chemotherapy drug targets.

To validate these hypotheses, we used 23 community cohesion scores of 50 breast cancer cell-line samples and their drug sensitivity data from GDSC. We observed that each of the 50 breast cancer cell-line samples also show the decreased community cohesion scores in the BrC19 (Figure 3B). More importantly, when all anti-cancer drugs in the GDSC were considered, the average IC50 values in the 50 breast cancer cell-lines were 294.14$\mathrm{\mu}$M (Figure 3C). However, the average IC50 values were significantly decreased to 35.087$\mathrm{\mu}$M when only the 28 drugs targeting the genes present in the BrC19 were considered (Wilcoxon rank-sum test, *P*-value = 2.55E-17) (Figure 3C). In addition, we observed that 40 and 10 breast cancer cell-lines samples show the highest connectivity loss score of KIF11 and AURKA, respectively (Figure 3D). Remarkably, we confirmed that the average IC50 values of the drugs targeting these individualized therapeutic targets in each cell-line sample decreased dramatically to 10.372$\mathrm{\mu}$M (Wilcoxon rank-sum test, *P*-value = 1.26E-10) (Figure 3C).

Given that the average IC50 values of the drugs targeting TYMS (e.g. Methotrexate), which are already approved chemotherapy drugs for breast cancer [38], were 64.155$\mathrm{\mu}$M in 50 breast cancer cell-line samples, these results highlights that the drugs targeting KIF11 and AURKA also could be effective individualized chemotherapy drugs for breast cancer. Interestingly, anti-cancer drugs targeting AURKA (e.g. Alisertib) are recently considered as potential therapeutic options [39].

### The community cohesion scores are superior to the previous individualized gene network estimation methods in terms of prioritizing individualized therapeutic targets

Furthermore, we wanted to compare our method with the gene expression profiles and the previous individualized gene network estimation methods in terms of prioritizing the individualized therapeutic targets. In the case of gene expression profiles, we normalized the gene expression profiles of the 50 breast cancer cell-line samples to z-scores and prioritized the individualized therapeutic targets which show the largest absolute value of z-scores in each sample. In the case of previous individualized gene network estimation methods, we estimated the individualized gene networks using SSN [20], LIONESS [21] and SWEET [22] methods. Then, we prioritized the individualized therapeutic targets based on the connectivity of the targets (hub genes) [20] or the shortest average path length of the targets to the hub genes in each individualized gene network [22].

As a result, we observed chemotherapy targets which are related to the cell cycle, such as CDK2, HDAC2 and BCL2, are predicted as the individualized therapeutic targets by the gene expressions and the previous individualized gene network estimation methods. Importantly, we confirmed that the drugs targeting the individualized therapeutic targets prioritized by them are significantly less effective than those prioritized by the community cohesion scores (*P*-value <0.001, Figure 3E, Supplementary Table S7 and Supplementary Figure S9). The community cohesion scores showed at least four times higher performance in predicting effective individualized chemotherapy targets on average.

These results indicate that the community cohesion scores could be a more effective tool in terms of predicting the individualized therapeutic targets compared with the gene expression profiles and the previous individualized gene network estimation methods. In addition, taken together, we can predict the individualized therapeutic targets based on the community cohesion scores for each individual patient in 2-fold. First, we can prioritize them through the druggable genes present in the community with the significantly decreased community cohesion scores. Then, we can pinpoint more individualized therapeutic targets by discovering the genes that greatly contribute to the community cohesion looseness in each individualized gene network.

### The community cohesion scores can be used to discover the disease subtypes and subtype-specific therapeutic targets

In the previous section, we showed that the community cohesion scores successfully prioritize the individualized chemotherapy targets. However, the chemotherapy could have serious toxic effects because they do not distinguish between normal cells and cancer cell [40]. Recently, targeted therapy which targets the genes that are abnormally expressed in a specific cancer subtype plays a critical role in reinforcing the conventional cytotoxic chemotherapy [11, 40, 41]. Hence, we next explored whether the community cohesion scores could discover the known breast cancer subtypes and prioritize the subtype-specific therapeutic targets. To this end, we used the community cohesion scores of 390 hormone receptor positive (ER+ and/or PR+, HER2-) breast cancer tissue samples (HR+ samples) and 99 triple negative breast cancer tissue (ER- and PR-, HER2-) samples (TNBC samples) from TCGA BRCA, which are the most distinct breast cancer subtypes based on immunohistochemistry (IHC) assay.

**Figure 4:**
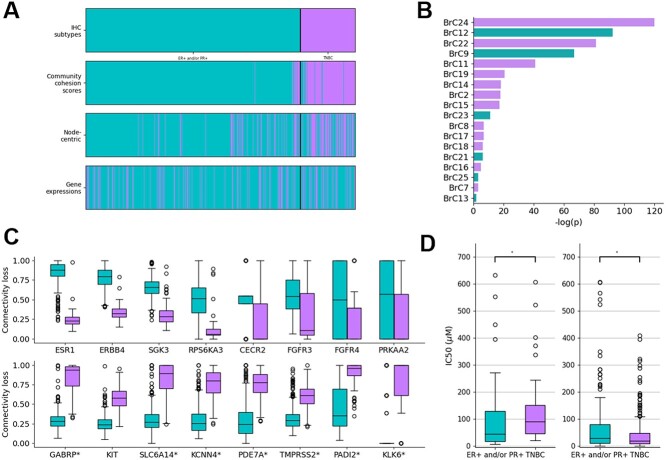
**Community cohesion scores as therapeutic biomarkers to discover individualized subtype-specific targets ** (A) The results of hierarchical clustering using the community cohesion scores, the node-centric community-based biomarkers and gene expression profiles were plotted. (B) The statistical significance (Wilcoxon rank-sum test) of community cohesion score difference compared between the two clusters was plotted. (C) The connectivity loss of the druggable genes present in the BrC12 and BrC24 in the individualized gene networks of the HR+ breast cancer samples and the TNBC breast cancer samples was compared. The druggable genes obtained from DrugBank are denoted using *. (D) The IC50 values of the anticancer drugs targeting ESR1 and KIT in the HR+ breast cancer cell line samples and TNBC cell-line samples were compared.

First of all, we divided the samples into two clusters using the community cohesion scores (hierarchical clustering, Supplementary Methods). As a result, 390 samples and 99 samples were clustered into the first and second clusters, respectively (Figure 4A). Notable, the clustering results were significantly similar with the IHC-based subtypes (Chi-square test, *P*-value = 4.56E-75) (Figure 4A). The first and second cluster were mostly composed of HR+ samples and TNBC samples, respectively. However, when the samples are clustered using the gene expression profiles, the distinct disease subtypes were not detected (Chi-square test, *P*-value = 0.339, Figure 4A).

Next, we explored the communities which are differently losing their community cohesions in the two clusters identified by the community cohesion scores. Among the 23 communities in the breast tissue, the community cohesion scores of the 18 communities were significantly different in the two clusters. Especially, the community cohesion scores of the BrC12 were significantly lower in the first cluster which is associated with HR+ breast cancer (Figure 4B). Interestingly, we found that the BrC12 contains ESR1, which is the target of endocrine therapy of HR+ breast cancer [42]. Furthermore, we more specifically explored whether ESR1 could be prioritized by the connectivity loss scores (Materials and Methods). For this, we compared the connectivity loss scores of eight druggable genes (CECR2, ESR1, ERBB4, FGFR3, FGFR4, PRKAA2, RPS6KA3 and SGK3) present in the BrC12 between the individualized gene networks of the samples belonging to the first and second clusters. As a result, we found that every eight druggable genes show significantly different connectivity loss scores in the two clusters (*P*-value <0.0001). Especially, ESR1 most significantly lose its connectivity in the first clusters (*P*-value = 8.92E-51, Figure 4C). We also confirmed that HR+ breast cancer cell-lines [43] were significantly more sensitive to the drugs targeting ESR1 than TNBC samples (*P*-value = 3.46E-04) using drug sensitivity data of GDSC (Figure 4D).

Similarly, we sought to identify new targeted targets for TNBC which does not have subtype-specific targeted therapy. We observed that the community cohesion scores of the BrC24 were significantly lower in the second cluster, which is associated with the TNBC (Figure 4B). According to the anti-cancer drug target information of GDSC, there are only one druggable gene (KIT) in the BrC24 and the connectivity loss scores of KIT was significantly larger in the individualized gene networks of the samples belonged to the second cluster (*P*-value = 1.24E-45, Figure 4C). In addition, we confirmed that the TNBC samples were significantly more sensitive to the drugs targeting KIT (*P*-value = 0.015, Figure 4D). Besides the druggable genes of the anticancer drugs in GDSC, we expanded the therapeutic targets using target information from DrugBank [44] and we discovered 15 more therapeutic targets in the BrC24. Among them, the connectivity loss scores of GABRP was the most significantly larger in the samples belonged to the second clusters (*P*-value = 2.62E-49, Figure 4C). Interestingly, there are growing pieces of evidence that GABRP could be a therapeutic target for TNBC [45–47].

In sum, we showed that the community cohesion scores are superior to the gene expression profiles in terms of discovering distinct subtypes of breast cancer. Furthermore, we successfully found the HR+ breast cancer specific targeted therapy target (ESR1) based on community cohesion scores which are significantly declined in the clusters which are associated with the HR+ breast cancer samples. Based on this result, we suggest new targeted therapy targets (e.g. KIT and GABRP) for TNBC, which has no subtype-specific therapeutic targets.

### The community cohesion scores can be used as prognostic makers to predict drug resistance and potential therapeutic targets for combination therapy

In the previous section, we showed that the community cohesion scores can successfully prioritize the subtype-specific therapeutic targets. Furthermore, we next turned to discover prognostic biomarkers which can predict drug resistance even in the same subtype of cancer using the community cohesion scores. To this end, we performed survival analysis of estrogen receptor positive (ER+) breast cancer patients treated with endocrine therapy, especially tamoxifen, to discover distinct prognostic subgroups. The endocrine therapy which targets ESR1 is considered as a first-line treatment for ER+ breast cancer patients [42]. However, the ER+ breast cancer patients show different prognosis due to the intrinsic and/or acquired resistance to the endocrine therapy. Therefore, more precise stratification method for the ER+ breast cancer is needed, which is able to predict the response to the endocrine therapy.

To this end, we measured the community cohesion scores of 181 tamoxifen-treated ER+ breast cancer tissue samples from GSE6532. Then, we divided the samples into two subgroups according to a threshold of the community cohesion scores in each community. We slightly incremented the threshold from zero to one (0.001) and find the optimal threshold which shows the most distinct prognostic difference between the two subgroups. We compared the 5-year recurrence free survival rate between the two subgroups using the Kaplan–Meier survival analysis and evaluated them through log-rank test (Supplementary Methods).

**Figure 5:**
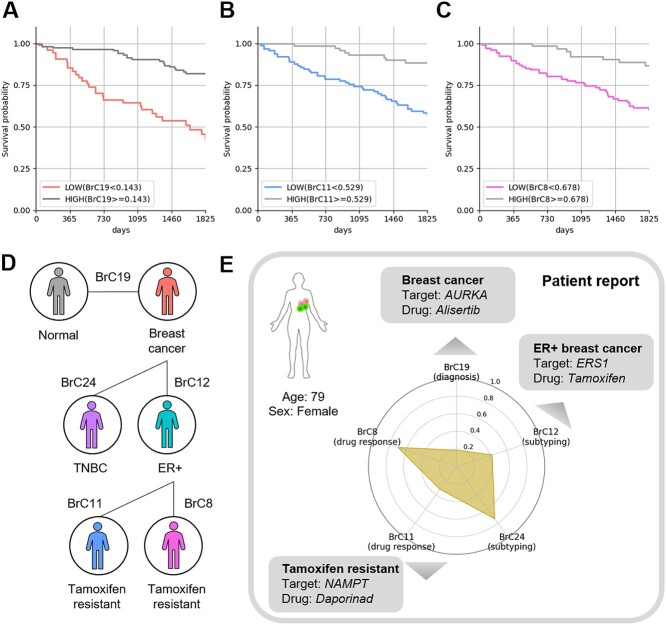
**Community cohesion scores as prognostic biomarkers to predict drug resistance** The Kaplan–Meier survival plots represented the prognostic subgroups of tamoxifen-treated ER+ breast cancer samples which are discovered by the community cohesion score thresholds in (A) BrC19, (B) BrC11 and (C) BrC8. (D) The diagnostic, therapeutic and prognostic biomarkers discovered by community cohesion scores in breast cancer were depicted. (E) The example patient report of utilization of the community cohesion scores in the clinical practice was illustrated. The human and cell figures were created with BioRender.com.

As a result, three community (BrC8, BrC11 and BrC19) successfully discovered the prognostic subgroups (*P*-value <0.001 and Supplementary Table S7). Among them, the prognostic difference between the two subgroups was most distinct when the samples were divided according to the community cohesion scores of the BrC19 (Figure 5A, threshold = 0.143, *P*-value = 1.37e-06). We observed that the samples which lose much community cohesions in the BrC19 show worse prognosis (*n* = 55). Interestingly, we already found that the BrC19 is associated with the molecular mechanisms of cancer (cell cycle) in the previous sections. It indicates that the serious dysfunction of the cell cycle processes could be regarded as the biomarker to endocrine therapy response prediction.

In addition to the BrC19, the BrC11 (Figure 5B, threshold = 0.529, *P*-value = 1.14E-04) and the BrC8 (Figure 5C, threshold = 0.678, *P*-value = 0.001) also successfully discover the distinct prognostic subgroups. Specifically, the samples that have the decreased community cohesion scores of the BrC11 (*n* = 102) and BrC8 (*n* = 111) showed the poor prognosis. These results indicate that the dysfunction of these communities neutralize the therapeutic effect of tamoxifen and the genes present in these community could be potential therapeutic targets to reduce resistances based on the individualized mechanisms of endocrine therapy resistance. For example, among the anti-cancer drug targets in the BrC11 (Supplementary Table S6), it has been known that high expression of NAMPT (nicotinamide phosphoribosyl transferase) confers the tamoxifen resistance [48] and recently it is discovered that combination therapy of NAMPT inhibition and antiestrogen is effective to reduce breast cancer metastasis [49]. Similarly, there are several pieces of evidence that targeting both ESR1 and RXRA (retinoid X receptor alpha) present in BrC8 (Supplementary Table S6) decreases the tamoxifen resistance [50].

Given these, the combination therapy considering the individualized mechanisms of endocrine therapy resistance could increase therapeutic effect of tamoxifen. For example, we suggested a use case of community cohesion scores as comprehensive biomarkers for precision oncology in clinical practice (Figure 5D and 5E). The individualized therapeutic targets of this case sample could be prioritized by finding the genes greatly contribute to the community cohesion looseness of disease-related (e.g. AURKA in the BrC19) or subtype-related community (e.g. ESR1 in the BrC12). In addition, the example sample showed the decreased community cohesion scores in the BrC11 and tamoxifen resistance is predicted. Hence, the gene present in the BrC11 could be considered as potential combinatorial therapy to reduce tamoxifen resistance.

## DISCUSSION

It has already been about 20 years since the concept of precision medicine appeared [51], and it is recently facing a new turning point with the advances of high-throughput technology and network biology. They have taken us a step closer to understanding the underlying mechanisms of the complex diseases. Now, it is time to apply them in clinical practices for realizing the precision oncology. To satisfy this demand, we proposed community cohesion scores as the community-based biomarkers for precision oncology. To this end, we first estimated the individualized gene network by identifying the individualized perturbed interactions which are deviated from co-expression patterns of the normal tissue. Next, in each individualized gene network, we measured the community cohesion scores which precisely quantify of the community ability to retain the normal interactions between the genes and their cellular functions by applying the network efficiency in each community.

However, there are several remaining challenges that should be considered in further studies. First, we determined the individualized perturbed interactions by evaluating whether the expression levels of the case sample are deviated from the co-expression patterns in the normal tissue. However, we did not consider whether the perturbations were due to strengthening or weakening the co-expressions. The co-expressions that are strengthened or weakened in the disease-context could have different effects in the disease states and considering them differentially could enhance our method. In addition, our method requires transcriptomics data obtained from tissue biopsy. However, it has been demonstrated that multi-omics approaches could suggest a new insight in terms of understanding the complexity of the diseases [52]. Interestingly, it has been revealed that the genetic mutations enriched in the protein–protein interactions surface [8, 53] have greater implications in the disease context. Additionally, there are several experimental and computational methods which can predict protein–protein interaction dynamics in specific biological context [54–56]. In this perspective, the addition of dynamic protein–protein interaction changes resulting from genetic mutations enables our method to more accurately depict individualized characteristics shown in the community cohesion looseness. Meanwhile, the biological interactions could be dyanamically changed in a specific context and regular biopsy sampling is essential to detect dynamic biological interactions. However, invaisve tissue biopsy preculdes the regular sampling. Recently, blood has been in spotlight as a window into health and diseases [57, 58] and there are several methods which can predict tissue-specific gene expressions using whole blood transcriptomes [59–61]. The inferred tissue-specific gene expressions from the noninvasive and regular whole blood samples enable our method to capture dynamics in the individualized gene networks.

Despite of these challenges, we validated that the community cohesion scores can be used as comprehensive biomarkers for precision oncology using breast cancer. We showed that the community cohesion scores are more robust biomarkers than single gene -based and node-centric community-based biomarkers through high reproducibility of breast cancer classification performance in the independent dataset. More importantly, we validated that the community cohesion scores successfully predict the individualized therapeutic targets in 2-fold; first by targeting the genes present in the community with the significantly decreased community cohesion scores and second by targeting the gene which greatly contribute to the community cohesion looseness (e.g. connectivity loss scores). We also showed that our method is superior to the previous individualized gene network estimation methods and gene expression profiles in terms of prioritizing the individualized therapeutic targets.

Furthermore, we demonstrated that the community cohesion scores successfully identified breast cancer subtypes (e.g. HR+ breast cancer and TNBC) and the subtype-specific therapeutic targets (e.g. ESR1 in the HR+ breast cancer) by exploring relatively decreased community cohesion scores in each subtype. Based on this result, we suggested potential therapeutic targets for TNBC, such as KIT and GABRP. Additionally, we showed that the community cohesion scores can be used to predict the drug response and suggested the potential combination therapies (e.g. NADPT or RXRA inhibitors) to reduce tamoxifen resistance for ER+ breast cancer. Overall, these results highlight that the community cohesion scores can be used as the therapeutic and prognostic biomarkers to precisely define the individual disease states and establish the treatment strategy. We hope that our method can contribute to accelerate the realization of precision oncology and can be applied to other complex diseases of which molecular context is tissue-specific, complex and heterogeneous (e.g. cardiovascular disease and liver cirrhosis) beyond the precision oncology.

**Key Points**

- Community cohesion looseness in the social networks increases the risk of diseases and morality. Community cohesion scores apply the concept of community cohesion to the gene networks to overcome the limitation of current single-gene based and node-centric community-based biomarkers.
- Community cohesion scores capture unique individualized characteristics of the community cohesion looseness by precisely quantifying the community ability to retain the interactions between the genes and their cellular functions in each individualized gene network.
- The individualized gene networks and community cohesion scores of 950 breast cancer case samples were used to validate that the community cohesion scores can be used as comprehensive biomarkers to predict the individualized drug targets and resistance for precision oncology.

## Supplementary Material

supplementary_materials_bbae175

Supplementary_table1_bbae175

Supplementary_table2_bbae175

Supplementary_table3_bbae175

Supplementary_table4_bbae175

Supplementary_table5_bbae175

Supplementary_table6_bbae175

Supplementary_table7_bbae175

Supplementary_table8_bbae175

## DATA AND MATERIALS AVAILABILITY

There are no primary data in the paper; all data are publicly available on online databases of GTEx (https://gtexportal.org/home), TCGA (https://portal.gdc.cancer.gov/), GEO (https://www.ncbi.nlm.nih.gov/geo/) and GDSC (https://www.cancerrxgene.org/). We have archived our code on GitHub at https://github.com/BISLshwang/Community-cohesion-scores.

## Author Biographies

**Seunghyun Wang** is a PhD student of the Department of Bio and Brain Engineering, Korea Advanced Institute of Science and Technology, Republic of Korea. Her research interests are bioinformatics, precision medicine and systems biology.

**Doheon Lee** is a professor of the Department of Bio and Brain Engineering, Korea Advanced Institute of Science and Technology, Republic of Korea. His research interests are bioinformatics, medical informatics and systems biology.
