# Supplementary material for: Community cohesion looseness in gene networks reveals individualized drug targets and resistance
Source: Brief Bioinform. 2024 Apr 15;25(3):bbae175. doi: 10.1093/bib/bbae175 (PMC11018546; doi:10.1093/bib/bbae175)
Supplement: supplementary_materials_bbae175 [file supplementary_materials_bbae175.docx]

**Supplementary materials**

**Supplementary Methods**

**Collecting the gene expression profiles of control samples**

We collected the gene expression profiles of control samples from the Genotype-Tissue Expression (GTEx) which is publicly available (Supplementary Figure S2) [1]. The version 8 data of GTEx consisted of 15,201 RNA-sequencing samples from 54 non-diseased tissue sites of 838 postmortem donors. We downloaded both gene read counts and TPMs (Transcripts Per Million) from the GTEx online portal (https://www.gtexportal.org/). In this study, we especially used 459 gene expression samples of “Breast – Mammary Tissue” as the control samples. Next, we downloaded the list of protein-coding genes from HGNC (https://www.genenames.org/) and remained only protein-coding genes [2]. We also regarded the genes of which median read counts are less than 10 across samples in each tissue sites as noises and excluded them. As a result, in the final gene expression profiles, the 459 control samples of the breast tissue and 15,581 genes were included.

**Collecting the gene expression profiles of case samples and the clinical data**

We collected gene expression profiles of case samples to construct the individualized gene networks. We used three large public databases, The Cancer Genome Atlas (TCGA, https://portal.gdc.cancer.gov/), Gene Expression Omnibus (GEO, https://www.ncbi.nlm.nih.gov/geo/), and Genomics of Drug Sensitivity in Cancer (GDSC, https://www.cancerrxgene.org/) to collect the case samples. Especially, we used Python library GEOparse (version 2.0.3) to query and retrieve data from GEO.

*TCGA BRCA*

We downloaded FPKM (Fragments Per Kilobase of transcript per Million) normalized RNA-sequencing data of 685 case samples (113 normal breast tissue and 572 breast cancer tissue samples) from TCGA BRCA [3]. Additionally, we obtained IHC (Immunohistochemistry) assay data (ER, PR, HER2) and cancer stage data (stage1~4) of 572 breast cancer tissue samples.

*GSE58135*

We downloaded FPKM (Fragments Per Kilobase of transcript per Million) normalized RNA-sequencing data of 134 case samples (50 normal breast tissue and 84 breast cancer tissue samples) from GSE58135 [4].

*GSE6532*

We downloaded RMA (Robust Multichip Average) normalized microarray data of 181 case samples (181 tamoxifen-treated ER+ breast cancer samples) from GSE6532 [5]. We additionally obtained clinical data of 181 case samples including recurrence free status and months.

*GDSC*

We downloaded RMA (Robust Multichip Average) normalized microarray data of 50 case samples (50 breast cancer cell-line samples) from GDSC [6]. We also obtained anti-cancer drug sensitivity data of the 50 breast cancer cell-line samples from GDSC1 and GDSC2 datasets. We collected IC50 values of 542 FDA-approved or investigational anti-cancer drugs associated with 329 therapeutic targets from the GDSC1 and GDSC2 datasets. If there is duplicate experimental conditions in both GDSC1 and GDSC2 (e.g., same cell-line and drug pairs), we used averaged IC50 values.

**Gene-set enrichment analysis**

To explore the cellular functions associated with each community, we performed gene-set enrichment analysis (Enrichr [7]) using the genes present in each community and four gene-set databases (Gene Ontology [8], KEGG [9], Reactome [10] and WikiPathways [11]). We used Python library GSEApy (version 0.14.0) to implement Enrichr.

**Normalizing the gene expression profiles of control and case samples**

We renormalized the gene expression profiles of the control and case samples to remove batch effect. We applied slightly modified median rank scores (MRS) [12-14] to normalize transcriptomics data (Supplementary Figure S3). The MRS requires one control dataset and we used the control samples from the GTEx as a control dataset. In the original MRS method, the median expression value of each gene in the control dataset is calculated. Instead, we sorted the gene expression values in ascending order in each control samples and median gene expression values of each rank were calculated. Then, the gene expression values in each control samples were replaced by the median gene expression value of each ranks. As the control samples, in each case samples, the genes were ranked according to the gene expression values. In the case of genes that are missed in the case samples, the ranks were replaced by the rank of control samples and the ranks of whole genes were re-ranked. Then, the ranks were replaced by the median gene expression values of each ranks in the control dataset. For example, in each breast cancer samples in the TCGA BRCA, the genes were ranked according to their expression values and the ranks were replaced by the median expression values of each rank calculated across the control samples of breast tissue from the GTEx dataset.

**Breast cancer classification**

To confirm that the community cohesion scores successfully represent the disease states, we trained classification models which discriminate the breast cancer samples from the normal tissue samples. As a training set, we used the 23 community cohesion scores of 113 normal tissue samples and 572 breast cancer samples obtained from TCGA BRCA. To make the balanced training and validation dataset, we randomly selected 113 samples out of the 572 breast cancer samples. Additionally, as an independent dataset, we measured the 23 community cohesion scores of 50 normal tissue samples and 84 breast cancer samples obtained from GSE58135. Like the training and validation dataset, we randomly selected the 50 out of the 84 breast cancer samples to make the balanced dataset. We trained 10 different logistic regression models and average AUROC scores were used to evaluate the prediction performance of the classification model.

Furthermore, we compared this prediction performance with those of the single gene-based and node-centric community-based biomarkers. Because the logistic regression models could be influenced by the number of features [15], we sorted out the top 23 differentially expressed genes (Supplementary Table S5) between the normal and breast cancer samples using limma [16] to make same number of features with the community cohesion scores and used the gene expression levels of them as the single gene-based biomarkers. In addition, for each sample, we made the node-centric community-based biomarkers by averaging the expressions levels of the genes present in each 23 communities. Like the community cohesion scores, we trained 10 different logistic regression models and average AUROC scores were used to evaluate the prediction performance of the classification model. We used Python machine learning library Scikit-learn (version 1.0.2) to train the logistic regression models.

**Hierarchical clustering for breast cancer subtyping**

To confirm that the community cohesion scores can be used to discovery the known breast cancer subtypes, we used the 23 community cohesion scores of 390 hormone receptor positive (ER+ and/or PR+, HER2-) breast cancer samples (HR+ samples) and 99 triple negative breast cancer (ER- and PR-, HER2-) samples (TNBC samples) from TCGA BRCA as input features for the hierarchical clustering. We used Python machine learning library Scikit-learn (version 1.0.2) for hierarchical clustering.

**The Kaplan-Meier survival analysis**

To evaluate that the community cohesion scores can be used as prognostic biomarkers to predict drug resistance, we used 23 community cohesion scores of 181 tamoxifen-treated ER+ breast cancer samples from GSE6532. We used recurrence free status and months data of the 181 samples to implement the Kaplan-Meier survival analysis [17]. We divided the 181 samples into two subgroups according to the specific threshold scores of each community and evaluated the significance of response difference between two subgroups using log-rank test [18]. We used Python library lifelines (version 0.27.3) for the survival analysis.

**Supplementary Figures**

**Supplementary Figure S1. The conceptualization of community cohesion scores**


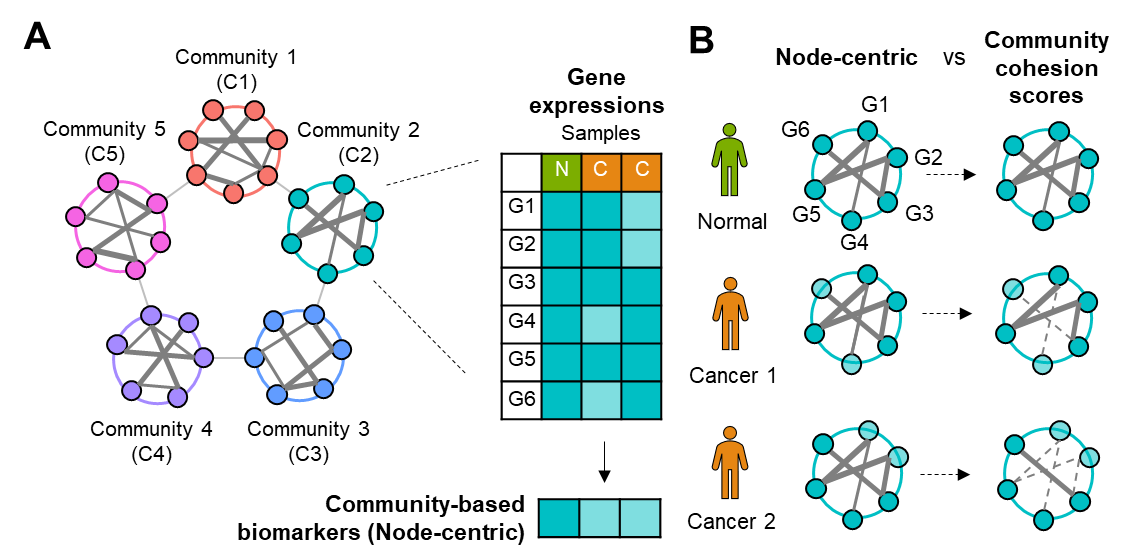


**Supplementary Figure S1. The conceptualization of community cohesion scores** (A) The current community-based biomarkers are generated by averaging expression levels of the genes present in the specific community. In the example of comparing normal (N) and cancer (C) samples, the community-based biomarkers can generate distinct feature which can discriminate cancer samples from the normal samples. On the other hand, there are no individual genes which are significantly highly or lowly expressed only in the cancer samples. (B) Beyond the current community-based biomarkers, community cohesion scores capture unique properties shown in the community cohesion looseness in the individualized gene networks and it allows more precise quantification of the community ability to retain the normal interactions between the genes and their cellular functions. For example, two cancer samples show identical scores when the current community-based biomarkers are used, but cancer 2 patients show abnormal expressions in the genes which interacts a greater number of genes and the functional declines could be more severe in this patient.

**Supplementary Figure S2. Constructing the normal tissue gene network (tissue-specific weighted co-expression network) and finding co-expressed community**


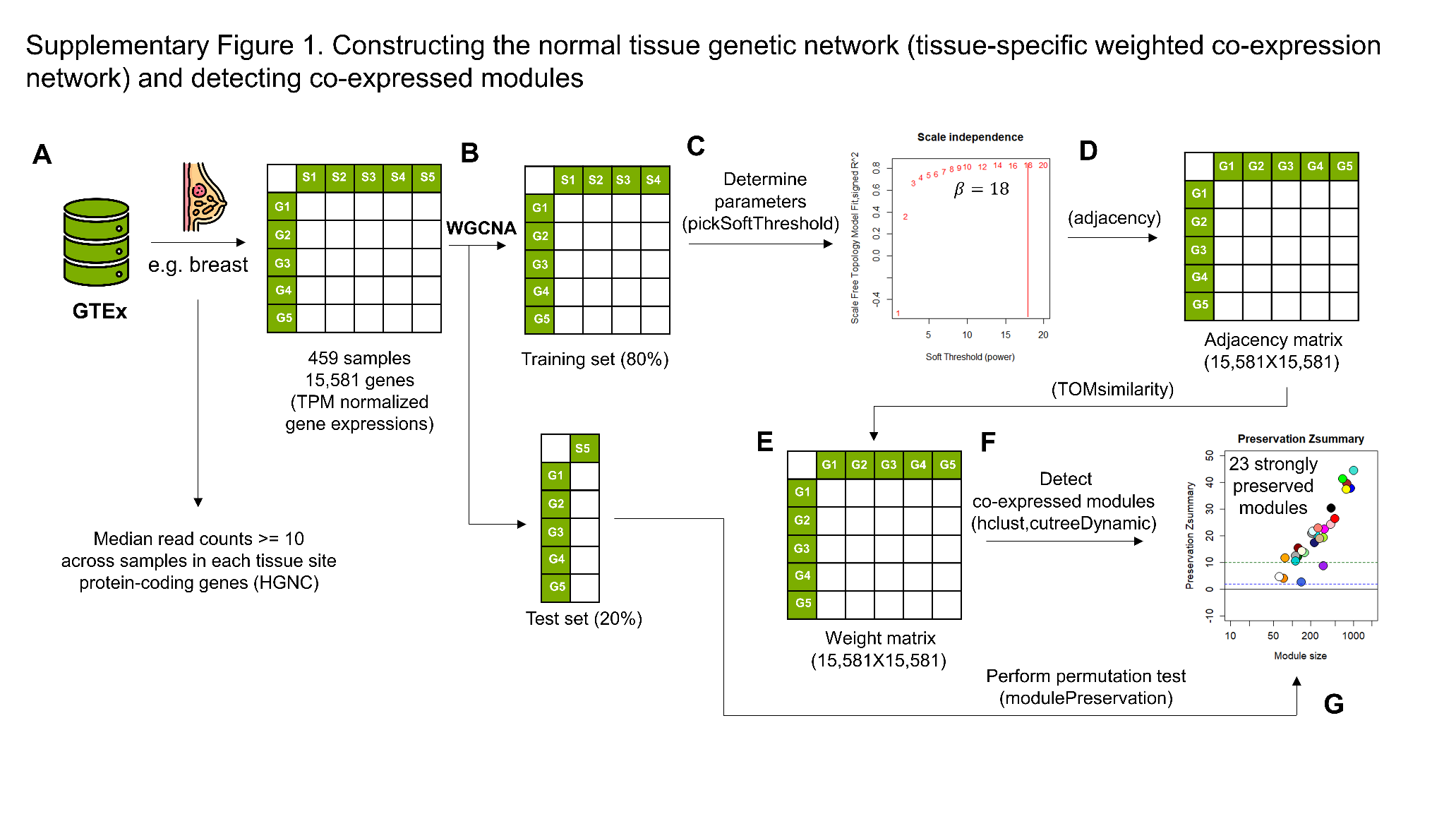


**Supplementary Figure S2. Constructing the normal tissue gene network (tissue-specific weighted co-expression network) and finding co-expressed communities** (A) We used the normalized gene expression profiles (gene TPMs) of the reference samples collected from the GTEx. (B) We divided the samples into training set (80%) and test set (20%). We used the training set (C) to determine parameters (pickSoftThreshold) and (D) to calculate adjacency matrix (adjacency) and € weight matrix (TOMsimilarity) to construct the weighted co-expression network. (F) Then, we detected the co-expressed modules which are highly interconnected with each other using the weight matrix as input features of hierarchical clustering (hclust, cutreeDynamic). (G) Moreover, permutation test was conducted to ensure the significance of each co-expressed module using the test set (modulePreservation). We only considered the co-expressed modules that are significantly preserved in the test set (preservation Zsummary > 10).

**Supplementary Figure S3. Gene expression normalization (Median Rank Score)**


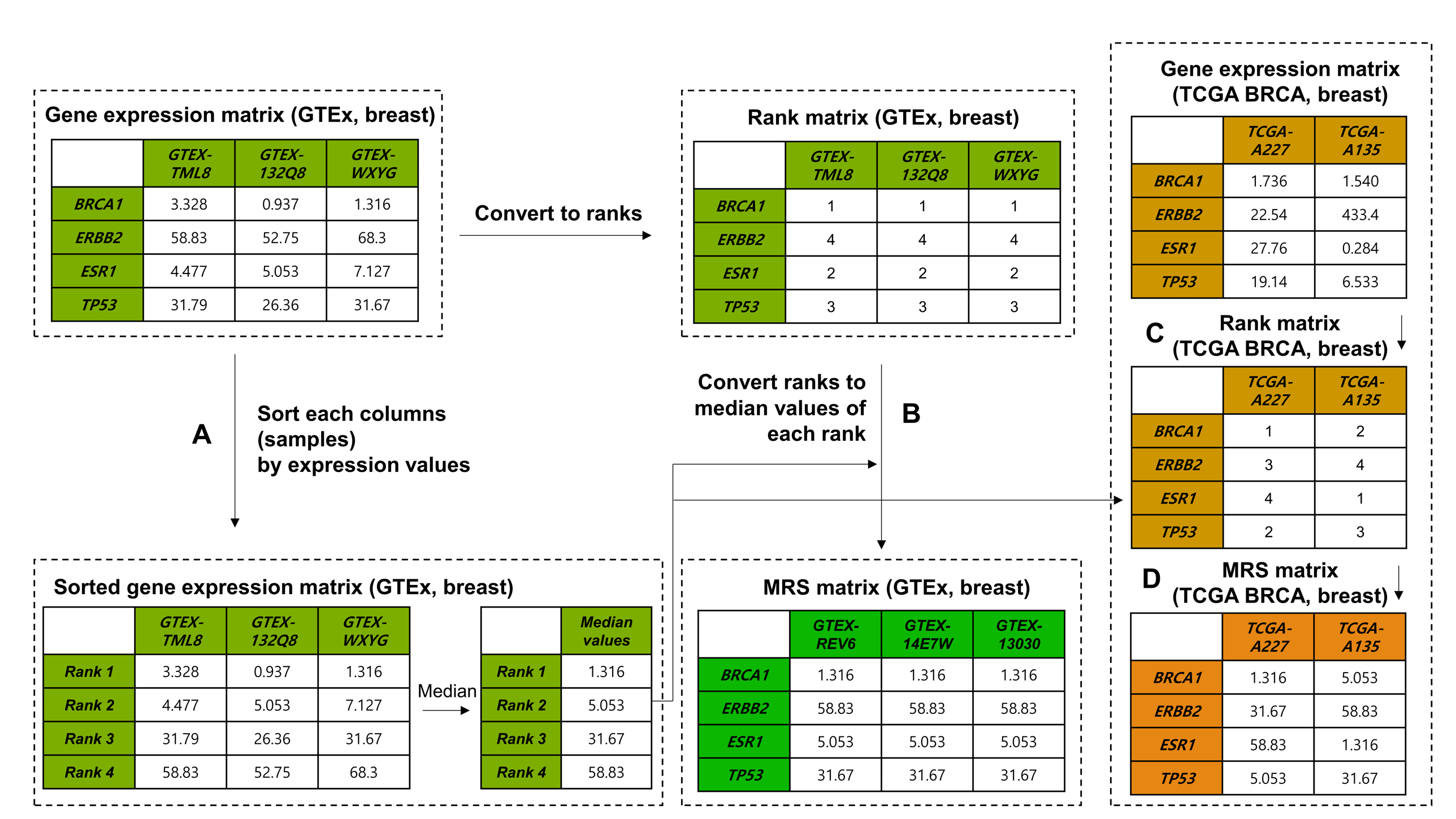


**Supplementary Figure S3. Gene expression normalization (Median Rank Score)** We renormalized the gene expression profiles of the reference and case samples to remove batch effect. We applied slightly modified median rank scores (MRS) to normalize transcriptomics data. The MRS requires one reference dataset and we used the samples from the GTEx as a reference dataset. In the original MRS method, the median expression value of each gene in the reference dataset is calculated. (A) Instead, we sorted the gene expression values in ascending order in each reference samples and median gene expression values of each rank were calculated. (B) Then, the gene expression values in each reference samples were replaced by the median gene expression value of each ranks. (C) As the reference samples, in each case samples, the genes were ranked according to the gene expression values. In the case of genes that are missed in the case samples, the ranks were replaced by the rank of reference samples and the ranks of whole genes were re-ranked. (D) Then, the ranks were replaced by the median gene expression values of each ranks in the reference dataset.

**Supplementary Figure S4. PCA plots of gene expressions normalized by median rank scores**


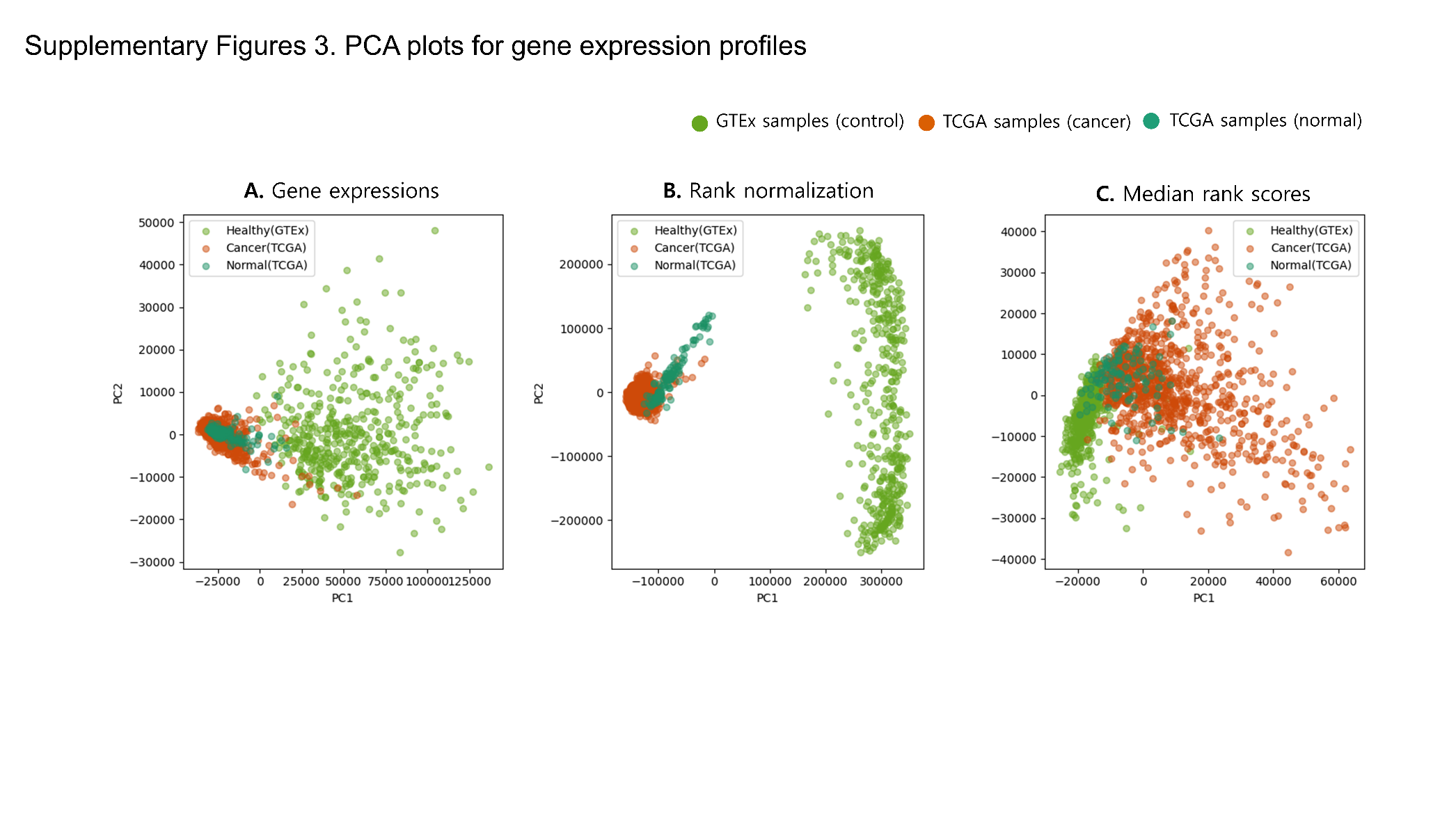


**Supplementary Figure S4. PCA plots of gene expressions normalized by median rank scores** PCA plots using (A) gene expressions, (B) Gene expressions normalized by rank, and (C) gene expressions normalized by median rank scores of normal breast tissue samples obtained from GTEx, normal breast tissue and breast cancer samples obtained from TCGA BRCA.

**Supplementary Figure S5. Modeling simple linear regressions of each interactions and measuring perpendicular distance distribution of the control samples**


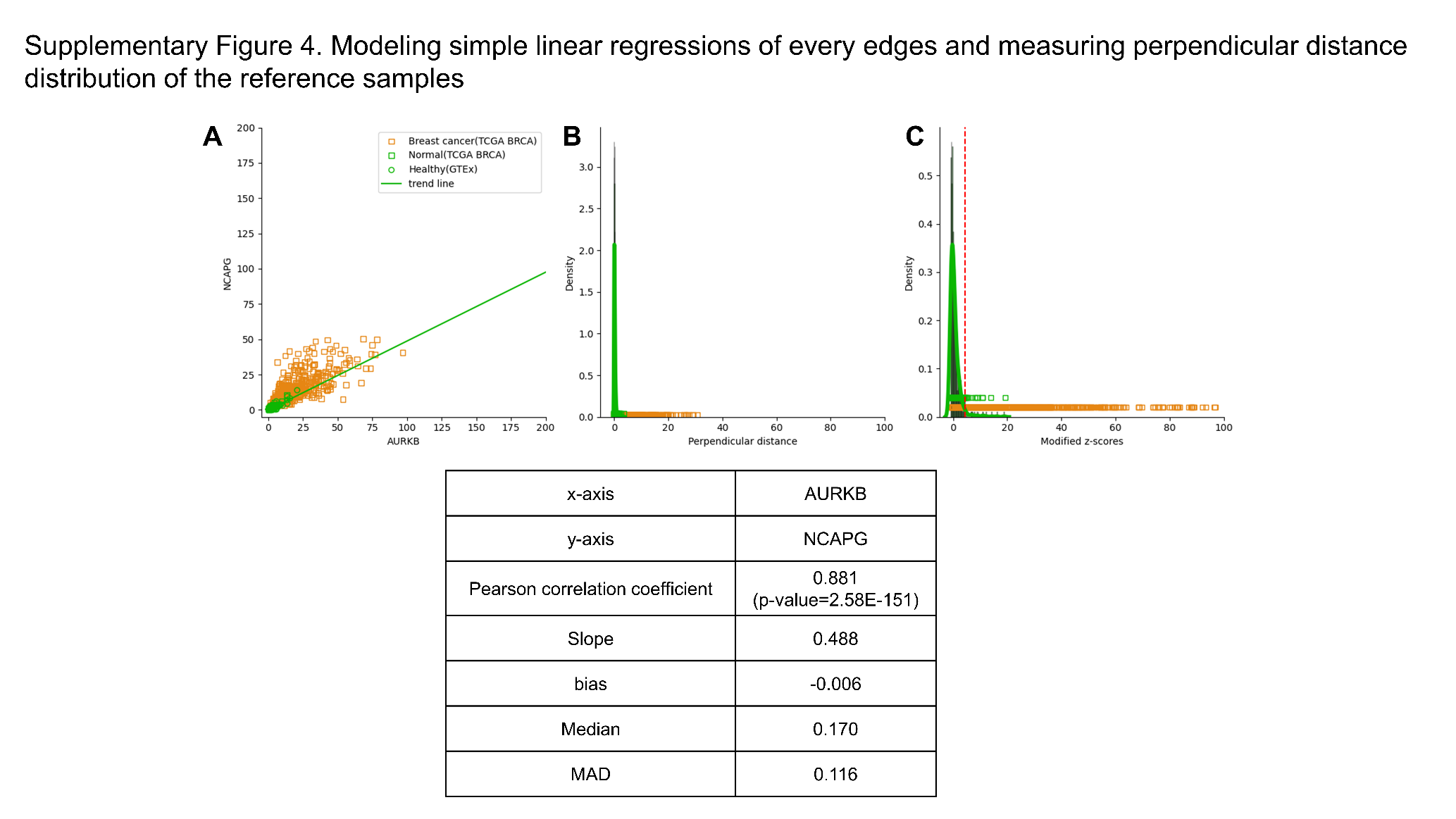


**Supplementary Figure S5. Modeling simple linear regressions of each interactions and measuring perpendicular distance distribution of the control samples** (A) For each interaction in the normal tissue gene network, we modeled the correlation of two genes (nodes) across the control samples through a simple linear regression. (B) Then, we made the perpendicular distance distribution of the control samples using the difference between the actual expression value and the linear regression models. Then, we measured the perpendicular distance of the individual case samples to evaluate whether the correlations between the correlations between the two genes are perturbed or not. (C) We converted them into modified z-scores using the median and the MAD of the perpendicular distribution made from the control sample. The interactions are considered as the individualized perturbed interactions if the results of one-tailed tests are statistically significant (p-value < 0.001).

**Supplementary Figure S6. Network efficiency and the community cohesion scores**


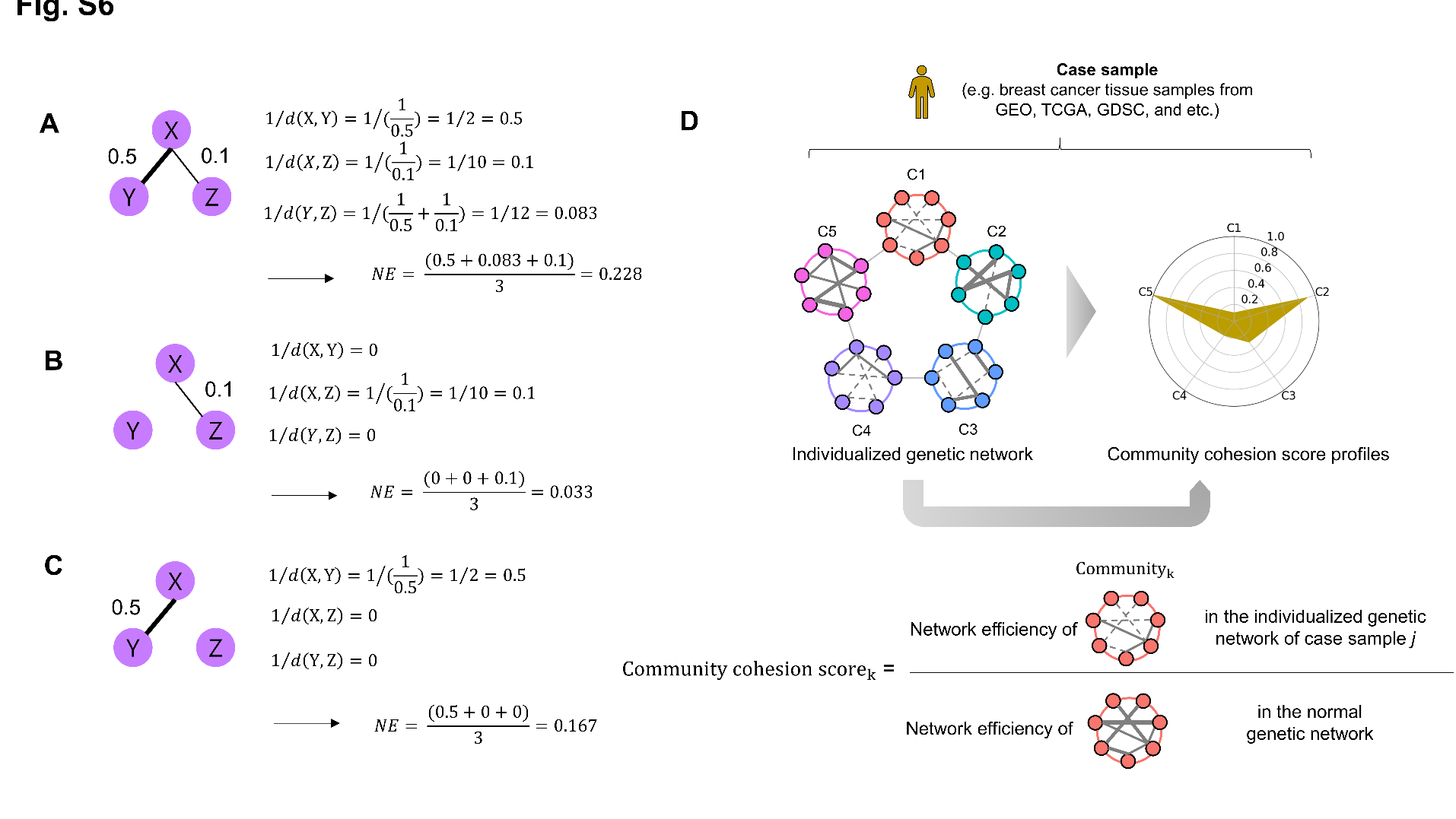


**Supplementary Figure S6. Network efficiency and the community cohesion scores** The network efficiency is a measure of the global complex network capacity to deliver information among nodes and allows a precise quantitative evaluation of the weighted network functioning. If we suppose that a network has two edges plotted in (A), the network in which the edge with larger weight is removed. (B) has dramatic decline in the network efficiency. (D) The community cohesion scores were defined as the decreased ratio of network efficiency of each community in each individualized gene network compared to those in the normal tissue gene network.

**Supplementary Figure S7. The heatmap of the community cohesion scores of TCGA BRCA samples**


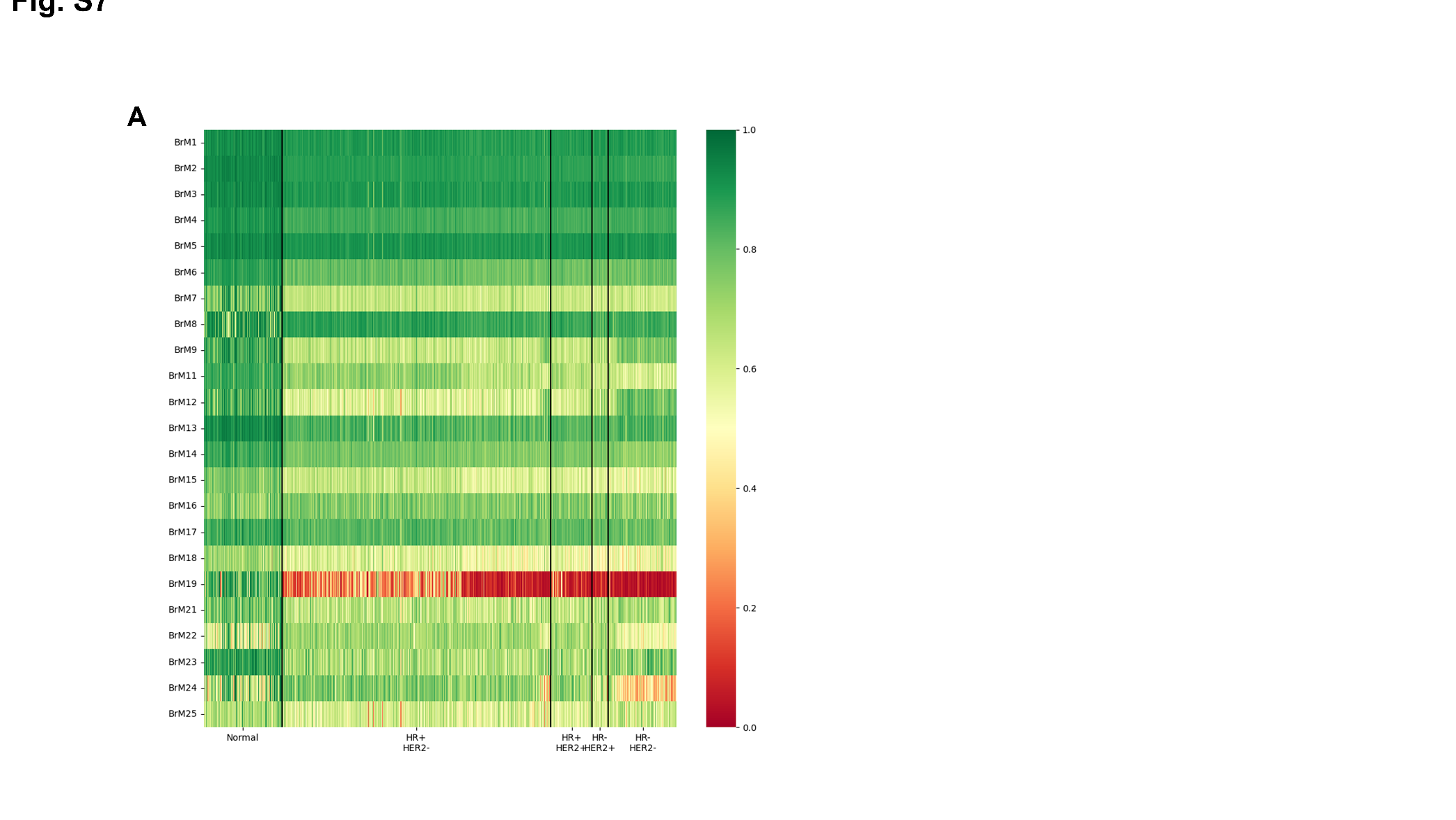


**Supplementary Figure S7. The heatmap of the community cohesion scores of TCGA BRCA samples** (A) The heatmap of the community cohesion scores of TCGA BRCA samples. The correlation between the size of community (the number of nodes) and the mean of community cohesion scores in (B) the normal breast tissue samples and (C) the breast cancer tissue samples.

**Supplementary Figure S8. The prediction performance (AUROC and accuracy) of breast cancer classification**


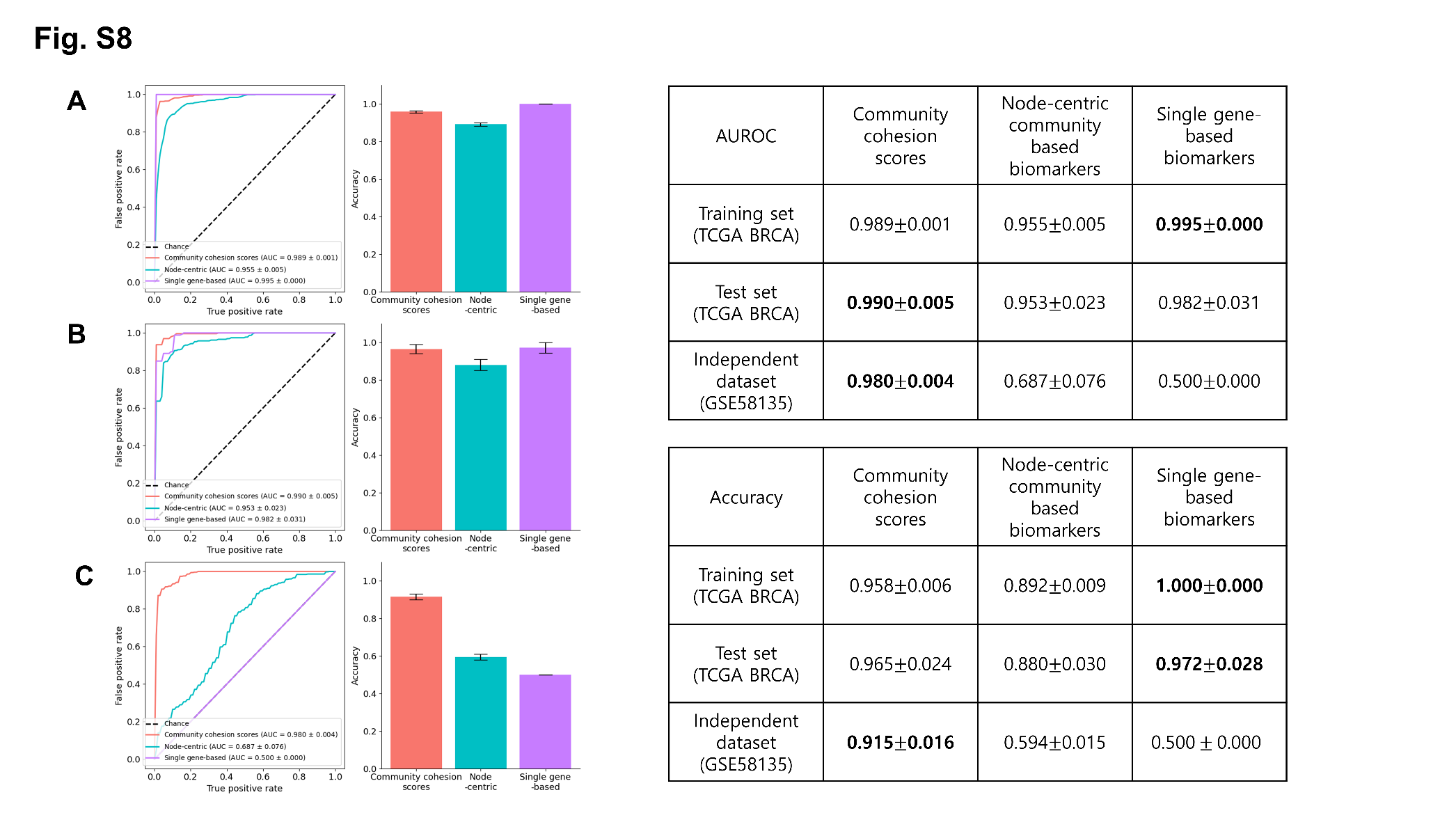


**Supplementary Figure S8. The prediction performance (AUROC and accuracy) of breast cancer classification** The classification performance (AUROC and accuracy) of the breast cancer samples and the normal samples in the balanced (A) training set (TCGA-BRCA), (B) validation set (TCGA-BRCA) and (C) independent test set (GSE58135)

**Supplementary Figure S9. The individualized therapeutic targets predicted by the gene expressions and the previous individualized gene network estimation methods**

**
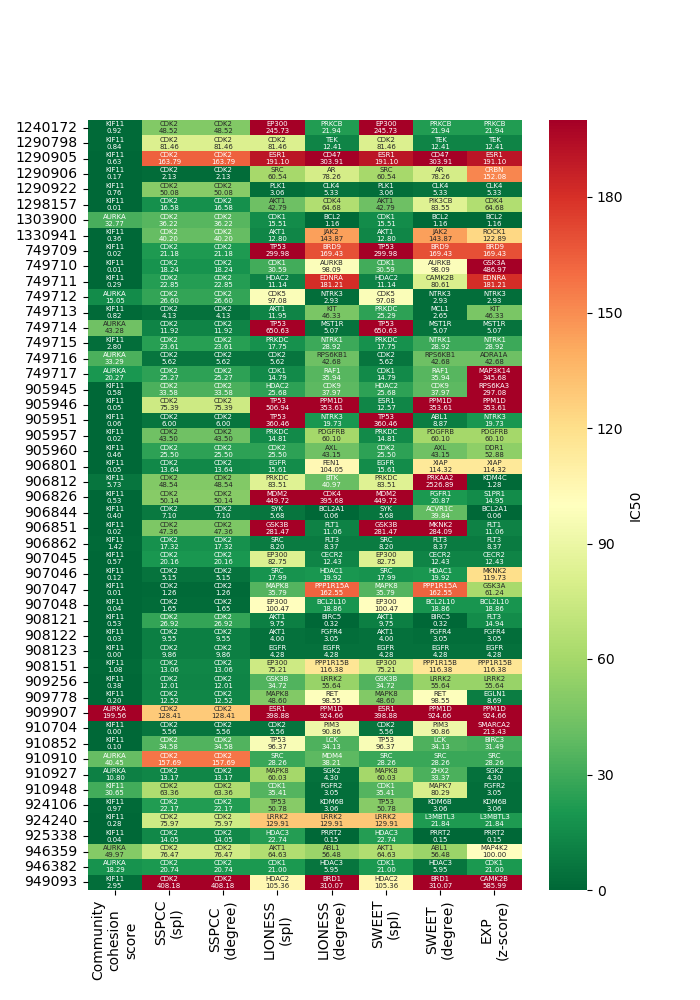
**

**Supplementary Figure S9. The individualized therapeutic targets predicted by the gene expressions and the previous individualized gene network estimation methods** The top 1 individualized therapeutic targets were predicted by the community cohesion scores, SSPCC [19], LIONESS [20], SWEET [21], and gene expressions (Z-scores). The y-axis indicates the 50 breast cancer cell-line obtained from GDSC and the individualized therapeutic targets and the IC50 values of the drugs targeting them in the corresponding cell-line are denoted as text and color, respectively.

**Supplementary Table captions**

**Supplementary table 1. The gene list of co-expressed communities in the normal breast tissue** The 27 co-expressed communities were detected in the normal breast tissue gene network. Among 15,581 genes in the control samples (normal breast tissue samples from GTEx), each of 8,977 genes belonged to one of the 27 communities and 6,604 gene were not assigned.

**Supplementary table 2. The preservation permutation test of the co-expressed communities** Among the 27 communities in the normal breast tissue gene network, only 23 highly preserved communities in the test set were considered in this study.

**Supplementary table 3. The statistics of the strongly preserved co-expressed communities** The number of nodes and edges before and after remaining the top 10% edges with the largest weights in each of 23 community

**Supplementary table 4. The gene-set enrichment analysis (Enrichr) results of BrM19** The gene-set enrichment analysis (Enrichr) of BrM19 using the genes present in the BrM19 and four databases (Gene Ontology, KEGG, Reactome, and WikiPathways)

**Supplementary table 5. The list of top 23 differentially expressed genes (limma) between normal breast and breast cancer tissue samples for breast cancer classification**

**Supplementary table 6. The anti-cancer drug and target list in BrC19, BrC12, BrC24, BrC8, and BrC11 according to the anti-cancer drugs and targets information**

**Supplementary table 7. The average IC50 values of the individualized therapeutic targets prioritized by community cohesion scores, previous individualized gene network estimation methods, and gene expressions** The individualized therapeutic targets of 50 breast cancer cell-line samples of GDSC were prioritized by the largest absolute value of z-scores of the gene expression profiles in each sample. In the case of previous individualized gene network estimation methods, the individualized gene networks were estimated using SSN, LIONESS, and SWEET methods. Then, the individualized therapeutic targets were prioritized based on the connectivity of the targets (hub genes) or the shortest average path length of the targets to the hub genes in each individualized gene network.

**Supplementary table 8. The survival analysis of ER+ breast cancer (log rank test)**

The significance of Kaplan-Meier survival analysis according to the threshold scores of each of 23 communities

**Reference**

1. Consortium G. The GTEx Consortium atlas of genetic regulatory effects across human tissues, Science 2020;369:1318-1330.

2. Povey S, Lovering R, Bruford E et al. The HUGO gene nomenclature committee (HGNC), Human genetics 2001;109:678-680.

3. Hoadley KA, Yau C, Hinoue T et al. Cell-of-origin patterns dominate the molecular classification of 10,000 tumors from 33 types of cancer, Cell 2018;173:291-304. e296.

4. Varley KE, Gertz J, Roberts BS et al. Recurrent read-through fusion transcripts in breast cancer, Breast cancer research and treatment 2014;146:287-297.

5. Loi S, Haibe-Kains B, Desmedt C et al. Definition of clinically distinct molecular subtypes in estrogen receptor-positive breast carcinomas through genomic grade, J Clin Oncol 2007;25:1239-1246.

6. Yang W, Soares J, Greninger P et al. Genomics of Drug Sensitivity in Cancer (GDSC): a resource for therapeutic biomarker discovery in cancer cells, Nucleic acids research 2012;41:D955-D961.

7. Chen EY, Tan CM, Kou Y et al. Enrichr: interactive and collaborative HTML5 gene list enrichment analysis tool, BMC bioinformatics 2013;14:1-14.

8. Ashburner M, Ball CA, Blake JA et al. Gene ontology: tool for the unification of biology, Nature genetics 2000;25:25-29.

9. Kanehisa M, Goto S. KEGG: kyoto encyclopedia of genes and genomes, Nucleic acids research 2000;28:27-30.

10. Jassal B, Matthews L, Viteri G et al. The reactome pathway knowledgebase, Nucleic acids research 2020;48:D498-D503.

11. Kelder T, Van Iersel MP, Hanspers K et al. WikiPathways: building research communities on biological pathways, Nucleic acids research 2012;40:D1301-D1307.

12. Tödling J, Spang R. Assessment of five microarray experiments on gene expression profiling of breast cancer. In: Proceedings of the 7th IEEE Annual International Conference on Computational Biology: April 10-13; Berlin, Germany. 2003.

13. Warnat P, Eils R, Brors B. Cross-platform analysis of cancer microarray data improves gene expression based classification of phenotypes, BMC bioinformatics 2005;6:1-15.

14. Lazar C, Meganck S, Taminau J et al. Batch effect removal methods for microarray gene expression data integration: a survey, Briefings in bioinformatics 2013;14:469-490.

15. Vabalas A, Gowen E, Poliakoff E et al. Machine learning algorithm validation with a limited sample size, PloS one 2019;14:e0224365.

16. Ritchie ME, Phipson B, Wu D et al. limma powers differential expression analyses for RNA-sequencing and microarray studies, Nucleic acids research 2015;43:e47-e47.

17. Kaplan EL, Meier P. Nonparametric estimation from incomplete observations, Journal of the American statistical association 1958;53:457-481.

18. Bland JM, Altman DG. The logrank test, Bmj 2004;328:1073.

19. Liu X, Wang Y, Ji H et al. Personalized characterization of diseases using sample-specific networks, Nucleic Acids Res 2016;44:e164.

20. Kuijjer ML, Tung MG, Yuan G et al. Estimating Sample-Specific Regulatory Networks, iScience 2019;14:226-240.

21. Chen H-H, Hsueh C-W, Lee C-H et al. SWEET: a single-sample network inference method for deciphering individual features in disease, Briefings in bioinformatics 2023;24:bbad032.
